# Real estate market and building energy performance: Data for a mass appraisal approach

**DOI:** 10.1016/j.dib.2015.11.027

**Published:** 2015-11-24

**Authors:** Pietro Bonifaci, Sergio Copiello

**Affiliations:** University IUAV of Venice, Italy

**Keywords:** Real estate market, Energy efficiency, Building energy performance, Mass appraisal, Hedonic price

## Abstract

Mass appraisal is widely considered an advanced frontier in the real estate valuation field. Performing mass appraisal entails the need to get access to base information conveyed by a large amount of transactions, such as prices and property features. Due to the lack of transparency of many Italian real estate market segments, our survey has been addressed to gather data from residential property advertisements.

The dataset specifically focuses on property offer prices and dwelling energy efficiency. The latter refers to the label expressed and exhibited by the energy performance certificate. Moreover, data are georeferenced with the highest possible accuracy: at the neighborhood level for a 76.8% of cases, at street or building number level for the remaining 23.2%.

Data are related to the analysis performed in Bonifaci and Copiello [1], about the relationship between house prices and building energy performance, that is to say, the willingness to pay in order to benefit from more efficient dwellings.

**Specifications Table**

**Table:** 

Subject area	*Economics*
More specific subject area	*Real Estate Market*
Type of data	*Table*
How data was acquired	*Survey*
Data format	*Raw*
Experimental factors	*Sample pretreatment as follows: sources with incomplete data have been discarded; variables have been measured in nominal, ordinal and interval scales; nominal and ordinal variables have been encoded into categories according to evidence arising from the data themselves*
Experimental features	*Records have been georeferenced according to different level of accuracy, specifically at the street or building number level when available, at the neighborhood level otherwise*
Data source location	*Padua, Italy*
Data accessibility	*Data are with this article*

**Value of the data**

• Raw data included here are suitable to be processed by means of a variety of analytical methods, both quantitative, as is the well-established multiple regression based hedonic price model [1,2], and mixed quali-quantitative, as is the rough set analysis [3].
• Georeferenced data lend themselves to be processed by means of spatial statistical analyzes, such as those based on interpolation models [4], [5], aiming to identify and represent the spatial distribution patterns of real estate values.
• The research branch devoted to evaluating the customers׳ willingness to pay for more efficient dwellings is growing interest, although the achieved outcomes are still conflicting and debated [6].

## 1 Data

The dataset features are as follows: 1,042 records, namely single unique properties, and 16 fields, namely property characteristics or other variables. Table 1 provides an insight on the measurement scales of the variables, as well as their coding system. The measurement scales have been defined according to the theoretical framework postulated by Stevens [7], who defined the following four fundamental scales: nominal (qualitative scale characterized by unordered categories); ordinal (expressing qualitative measures by means of a ranking); interval (expressing quantitative measures by means of successive values); ratio (expressing quantitative measures with a true zero point)*.*

Within the dataset, coding systems adopted for the variables based upon nominal and ordinal scales does not rely on a preconceived scheme, instead they are driven by the information usually available in residential property advertisements.

## 2 Experimental design, materials and methods

### 2.1 Experimental design

The city of Padua locates in Northern Italy and has been chosen for the survey because of its dynamic real estate market. According to cadastral statistics [8], in 2013 the city was accounted for a large building stock with a total of 174,250 real estate units, mainly composed by dwellings (114,702 units corresponding to 65.8% of total), manufacturing buildings (12,654 units; 7.3%), retail stores (9,077 units; 5.2%) and offices (6,486 units; 3.7%).

The dynamism of the local real estate sector is further witnessed by the amount of transactions recorded in the national observatory on the property market [9]. Padua has been accounted for 1,618 transactions of residential properties in 2013, namely 1.4% of housing stock. Although the ratio was considerably higher just before the 2008 crisis (3,271 dwelling transactions in 2007, 2.9% of housing stock), and in the meanwhile a turning point has been observed in the price trend, the empirical evidence still confirms it may be included among well-performing markets [10], [11], [12].

**Fig. 1:**
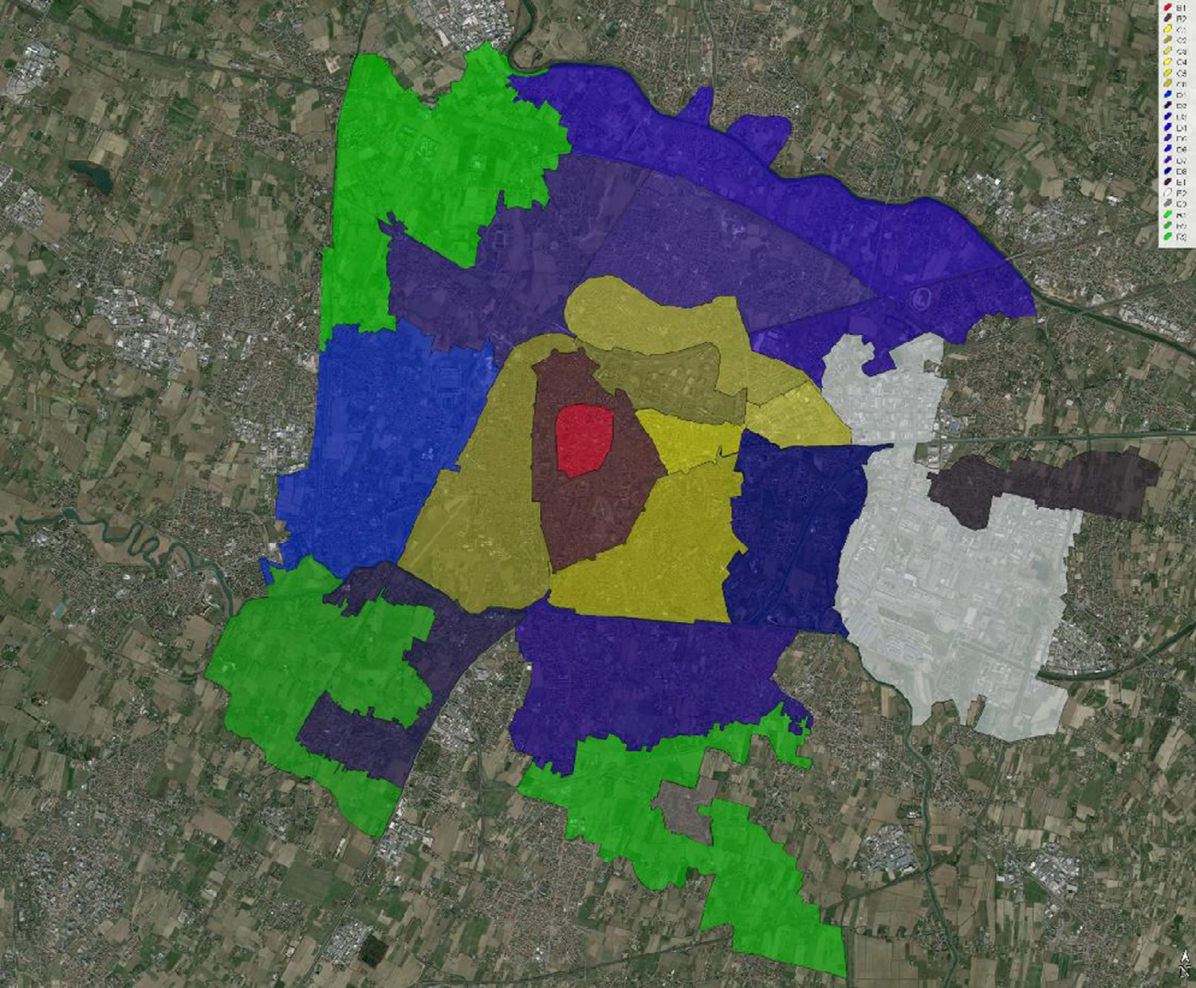
The city of Padua and the zones of the real estate market monitor.

The maintenance of a real estate market monitor is among the commitments undertaken by the Italian Revenue Agency. This monitor provides a full coverage of the national territory, by dividing it into homogeneous zones. The zones are identified by means of codes composed by a progressive number following a capital letter. In turn, the letters are assigned according to five kinds of locations: B stands for downtown areas; C for inner-ring areas; D for outer-ring areas; E for the outskirts and R for the rural areas. Usually, C, D, and E zones encompass both residential and industrial settlements. The real estate market monitor divides Padua into 22 zones, as represented in Fig. 1. The perimeter of the zones is also provided in the Supplementary materials within this article as a separate kmz file, which can be read with the Google Earth application.

Owing to the growing interest in building energy efficiency, several regulations have been enacted in Italy since the mid-seventies. When the Law 373/1976 entered into force, it constituted the first attempt to impose constraints on the building energy consumptions. Subsequently, a major enhancement to deal with the energy saving issue was represented by the Law 10/1991. Most recently, trying to meet the increasing consumers׳ demand for market transparency, the Decree-Law 63/2013 – implementing the European Directive 2010/31/EU – has introduced the legal obligation to include energy-related information within real estate advertisements. The data which are to be exhibited refer to the global energy performance index and the consequent energy label, as they are reported in the mandatory energy performance certificate [13]. Nonetheless, during the first time span in which the new regulation was in force, real estate advertisements were allowed to omit the value of global energy performance index. This is the reason why the dataset mainly focuses on the energy labels.

The Italian coding system of energy label relies on eight classes, from A+, which is the best one, to G, the worst. Each class correlates to the energy performance index, depending on the climate zone. The city of Padua is characterized by a yearly amount of Heating Degree Days (HDD, see European standard EN ISO 15927-6) equal to 2,383. Therefore, according to the classification of the national territory (Presidential Decree 412/1993), it falls within the E climatic zone (HDD within the range between 2,100 and 3,000). For this zone, Table 2 shows the energy performance index thresholds, for winter heating, corresponding to the aforementioned energy labels. During the survey, no cases of dwellings classified as A+ were found, hence they are missing in the dataset.

The data provided here are useful to investigate the relationship between the energy performance of housing and the expected selling price by owners. Such a relationship is predicted to be positive by the literature; nevertheless, its functional form – linear or not – and magnitude are still debated. Moreover, georeferenced data allow to analyze the potential spatial patterns of both real estate prices and building energy performances [16].

### 2.2 Method

The data have been gathered within a research branch devoted to exploring the relationships among building energy performances, the prices in the real estate market, the costs in the construction sector and the provision of affordable dwellings [17], [18]. The survey was conducted during the time span from 2013 April to July. A total of 1,042 property advertisements have been accessed, filtering those published on dedicated websites by both private sellers and real estate agents. Only residential properties have been considered.

Main real estate advertisement-posting websites have been inspected daily during the survey period. They have been cross-checked to avoid considering the duplicate items. When an old advertisement has been found to have been posted again without changes, only initial data have been kept. On the contrary, reissued advertisements with changes led to discarding the prior ones, hence oldest data have been replaced by the newest.

Advertisements are usually concise; moreover, they may contain different kinds of information and make use or not of supporting materials, such as pictures of interiors and exteriors, or floor plans. The sample here provided includes only records characterized by complete information with regard to the previously mentioned variables (see Table 1). On the contrary, advertisements with missing data have been discarded.

Based on the location declared in the advertisements, data have been georeferenced but they are characterized by heterogeneous levels of accuracy. The sample implements the exact geographical position for dwellings whose advertisement contained the full address. The dwellings whose advertisement provided the street name, but not the building number, have been located by referring to the midpoint of the self-same street. Otherwise, advertisements providing only succinct information about the location have entailed the need to refer to the midpoint of neighborhoods.

## Figures and Tables

**Table 1:** Variables, measurement scales and coding systems.

*Code*	*Variable*	*Measurement scale*	Coding system
*ID*	*Identifier*	*Interval*	
*LAT*	*Latitude*	*Interval*	
*LON*	*Longitude*	*Interval*	
*ACC*	*Accuracy of georeferencing*	*Ordinal*	1: *Building number level*; 2: *Street level*; 3: *Neighborhood level*
*ZMM*	*Zones in the real estate market monitor*	*Nominal*	*C2*; *C3*; *C5*; *C6*; *C7*; *D1*; *D2*; *D3*; *D4*; *E1*; *E2*; *R1* (a)
*MC*	*Age and maintenance conditions*	*Ordinal*	1: *New building*; 2: *Aged building in good conditions*; 3: *Aged building in ordinary conditions*
*TYP*	*Dwelling typology*	*Nominal*	1: *Flat*; 2: *Detached house*
*FL*	*Floor level*	*Interval*	0 *for detached houses as well as for flats on the ground floor*
*RM*	*Number of rooms*	*Interval*	
*BR*	*Number of bathrooms*	*Interval*	
*PRK*	*Parking facilities*	*Interval*	0: *Absent*; 1: *Parking lot*; 2: *Single garage*; 3: *Double garage*
*AMT*	*Amenities: terraces*	*Nominal (dummy)*	0: *Absent*; 1: *Present*
*AMG*	*Amenities: garden*	*Nominal*	0: *Absent*; 1: *Commonly owned garden*; 2: *Privately owned garden*
*EPL*	*Energy performance label*	*Ordinal*	*A* (*nearly best energy class*); *B*; *C*; *D*; *E*; *F*; *G* (*worst energy class*)
*SRF*	*Surface*	*Ratio*	*Saleable square meters*
*PRC*	*Price*	*Ratio*	*Total offer price in euros*

(a) For spatial distribution, see the following Fig. 1 and refer to supplementary materials.

**Table 2:** Energy labels and energy performance index threshold for an E climatic zone.

*Energy label*	*Energy performance index threshold* (kW h/m^2^ y)	*Reference*
*A+*	<14	*Passive house*
*A*	<29	*Low consumption building*
*B*	<58	
*C*	<87	*Minimum standard requirement for new construction* (a)
*D*	<116	*Medium to high consumption building*
*E*	<145	
*F*	<175	*Very high consumption building*
*G*	≥175	

(a) According to a mean compactness ratio of 0.97 for a detached house-like typology [14], the compactness ratio is defined as “the ratio of the envelope surface exposed to the external environment over the conditioned volume” [15, p. 849].
